# Profiles of Comprehensive Morphological Phenotypes and Outcomes in Functional Mitral Regurgitation

**DOI:** 10.1016/j.jacasi.2025.11.014

**Published:** 2026-01-21

**Authors:** Cheng-Wei Lien, Wei-Jyun Wang, Kuan-Yu Lai, Jih-Chang Yu, Chung-Yen Lee, Jui Wang, Meng-Han Tsai, Chung-Lieh Hung, Shih-Hsien Sung, Hsien-Li Kao, Yi-Lwun Ho, Masaaki Takeuchi, Li-Tan Yang

**Affiliations:** a Division of Cardiology, Department of Internal Medicine, National Taiwan University Hospital, Hsin-Chu Branch, Hsin-Chu, Taiwan; b Department of Internal Medicine, National Taiwan University Hospital, Taipei, Taiwan; c Department of Statistics, National Taipei University, Taipei, Taiwan; d Institute of Epidemiology and Prevention Medicine, National Taiwan University, Taipei, Taiwan; e Health Data Research Center, National Taiwan University, Taipei, Taiwan; f Cardiovascular Division, Department of Internal Medicine, MacKay Memorial Hospital, Taipei, Taiwan; g Division of Cardiology, Department of Internal Medicine, Taipei Veterans General Hospital, Taipei, Taiwan; h Telehealth Center, National Taiwan University Hospital, Taipei, Taiwan; i Department of Laboratory and Transfusion Medicine, Hospital of University of Occupational and Environmental Health, School of Medicine, Kitakyushu, Japan

**Keywords:** functional mitral regurgitation, heart failure, transthoracic echocardiography

## Abstract

**Background:**

Current guidelines do not include phenotypical classifications in functional mitral regurgitation (FMR).

**Objectives:**

The study sought to classify FMR into 3 morphological phenotypes and assess determinants of mortality.

**Methods:**

We included patients with ≥moderately severe FMR from 2 tertiary centers. FMR was classified as ventricular functional mitral regurgitation with symmetrical tethering (VFMRst), ventricular functional mitral regurgitation with asymmetrical tethering (VFMRat), or atrial functional mitral regurgitation (AFMR). The primary composite endpoint was cardiovascular death and death equivalent (ie, left ventricular assist device implantation or heart transplantation).

**Results:**

Of 699 patients (70 years [Q1-Q3: 60-79 years], 46% female), VFMRst was the most prevalent (54%). VFMRat exhibited larger mitral regurgitation vena contracta (7.7 mm [Q1-Q3: 7-8.7 mm] vs 7.3 mm [Q1-Q3: 6.7-8.2 mm]) but smaller left ventricular end-diastolic volume index (100 mL/m^2^ [Q1-Q3: 83-128 mL/m^2^] vs 106 mL/m^2^ [Q1-Q3: 88-132 mL/m^2^]) than VFMRst (both *P* ≤ 0.037). AFMR with hamstrung posterior mitral valve (MV) exhibited larger left atrial volume index (96 mL/m^2^ [Q1-Q3: 77-145 mL/m^2^] vs 78 mL/m^2^ [Q1-Q3: 63-97 mL/m^2^]) and mitral regurgitation vena contracta (7.5 mm [Q1-Q3: 6.8-8.6 mm] vs 7.0 mm [Q1-Q3: 6.4-8.2] mm) than AFMR with central MV malcoaptation (both *P* ≤ 0.010). At a median follow-up of 2.6 years (Q1-Q3: 0.9-5 years), 193 primary endpoints occurred. Five-year event-free survival was higher in AFMR (71% ± 4%) than VFMRst (64 ± 3%) and VFMRat (63 ± 4%) (*P* ≤ 0.030), but adjusted outcomes were similar across groups (all *P* ≥ 0.139). Besides conventional markers, multivariable models demonstrated that low diastolic blood pressure (DBP), left atrial reservoir strain (LASr), and left ventricular longitudinal strain from the apical 4-chamber view (A4C-LVLS) independently linked to primary endpoints (all *P* ≤ 0.043). Adjusted spline curves showed increased primary endpoint risk with DBP ≤73.7 mm Hg, LASr ≤14.8%, and A4C-LVLS ≤9.3%.

**Conclusions:**

VFMRat exhibited more disproportionate features than VFMRst. AFMR with hamstrung posterior MV may indicate a more advanced disease stage. Morphological phenotypes alone were not associated with adjusted outcomes. LASr, A4C-LVLS, and DBP may assist in risk stratification in FMR.

Functional mitral regurgitation (FMR) is associated with high mortality, even in mild cases.^1^ Nevertheless, the management of FMR is considerably challenging. The COAPT (Cardiovascular Outcomes Assessment of the MitraClip Percutaneous Therapy for Heart Failure Patients with Functional Mitral Regurgitation) and MITRA-FR (Percutaneous Repair with the MitraClip Device for Severe Functional/Secondary Mitral Regurgitation) trials explored the benefits of mitral transcatheter edge-to-edge repair (M-TEER) compared with guideline-directed medical therapy.^2^^,^^3^ The divergent results of these trials have led researchers to theorize that FMR has different phenotypes,^4^ which have not yet been addressed in the latest guidelines.^5^^,^^6^ Accordingly, the concept of proportionateness has been widely adopted to explain the heterogeneous nature of FMR.^7^ However, the RESHAPE-HF2 (Randomized Investigation of the MitraClip Device in Heart Failure: Second Trial in Patients with Clinically Significant Functional Mitral Regurgitation) trial challenged this concept by showing consistent benefits of M-TEER in moderate to severe FMR with smaller effective regurgitant orifice area (EROA).^8^ Therefore, the complexity of FMR remained unsolved. Yet, few studies have focused on the morphological differences in FMR. Three major phenotypes, ventricular functional mitral regurgitation (VFMR), ischemic functional mitral regurgitation (IFMR), and atrial functional mitral regurgitation (AFMR), have been proposed to classify FMR based on the pathophysiological perspective.^9^ The prognostic values of novel indicators such as left atrial (LA) and left ventricular (LV) strain have been reported for FMR, although these have not yet been tested from a phenotypical standpoint.^10^^,^^11^

In light of these findings, our study aimed to: 1) compare profiles and outcomes of FMR phenotypes using comprehensive morphological classifications; and 2) explore determinants of outcomes and identify potential clinical cutoffs.

## Methods

### Study population and clinical data

This retrospective study included consecutive patients ≥18 years of age with moderately severe or severe FMR and a transthoracic echocardiogram (TTE) performed between January 2010 and July 2022 at 2 tertiary referral centers (National Taiwan University Hospital and National Taiwan University Hospital Hsin-Chu Branch). All TTEs were de novo reviewed to determine eligibility. Detailed exclusion criteria are shown in Supplemental Figure 1. All study participants underwent comprehensive cardiology and/or cardiovascular surgery evaluations within 30 days of TTE. Baseline NYHA functional class, comorbid conditions, medications, and surgical procedures (mitral valve [MV] repair/replacement, coronary artery bypass grafting [CABG], extracorporeal membrane oxygenation, left ventricular assist device (LVAD), and heart transplantation [HTx]) were independently and prospectively recorded, and were meticulously abstracted from electronic medical records or paper charts. The Charlson Comorbidity Index was computed. Both Institutional Review Boards approved this study (202102058RINA and 111-190-F); informed consent was waived. Our study adhered to the STROBE (Strengthening the Reporting of Observational Studies in Epidemiology) reporting guideline.

### Echocardiography and FMR phenotypes

In patients with multiple TTEs, the first eligible study was used for analysis. TTEs were performed by trained sonographers using commercially available echo systems. All TTEs were de novo reviewed and reassessed the following: LV dimensions/volumes (biplane disk-summation method), LA volume, MR severity, mitral annular dimensions (measured from apical 4-chamber [A4C] view at diastole where the MV opening was largest), and right ventricular (RV) function. Left ventricular ejection fraction (LVEF) was derived from volumetric method. RV function was assessed by fractional area change (FAC) and an overall eyeball assessment (0: normal; 1: mildly reduced; 2: moderately reduced; 3: moderately to severely reduced; 4: severely reduced). For MR severity, an integrated, comprehensive approach including quantitative (EROA derived from proximal isovelocity surface area method) and semi-quantitative measures (vena contracta [VC] width measured in A4C, pulmonary vein reversal) was used.

We classified FMR into 3 major morphological phenotypes in accordance with the study by Reddy et al^9^: 1) for VFMR, a central MR jet arising from symmetrical MV tethering due to equal displacement of 2 papillary muscles, mainly caused by global LV dilation or wall motion abnormality (WMA); 2) for IFMR, a posteriorly directed MR jet arising from asymmetrical MV tethering with more tethering of the posterior leaflet and an overriding anterior leaflet, which mostly derived from inferolateral WMA or uneven LV remodeling; and 3) for AFMR, MR caused by mitral annular dilatation and lack of MV central coaptation owing to LA dilatation. As ischemic heart disease can also cause VFMR, we modified nomenclatures to avoid confusion: 1) VFMR was designated ventricular functional mitral regurgitation with symmetrical tethering (VFMRst); and 2) IFMR was designated ventricular functional mitral regurgitation with asymmetrical tethering (VFMRat).^12^ An AFMR subset caused by isolated hamstrung posterior mitral leaflet (PML) due to atrial remodeling,^13^ which led to posterior jet being classified as AFMR with hamstrung PML (Central Illustration). The first author (C.-W.L.) de novo reviewed all TTEs to discern the 3 MR phenotypes, and C.-W.L. and L.-T.Y. discussed ambiguous cases to reach consensus.

**Central Illustration:**
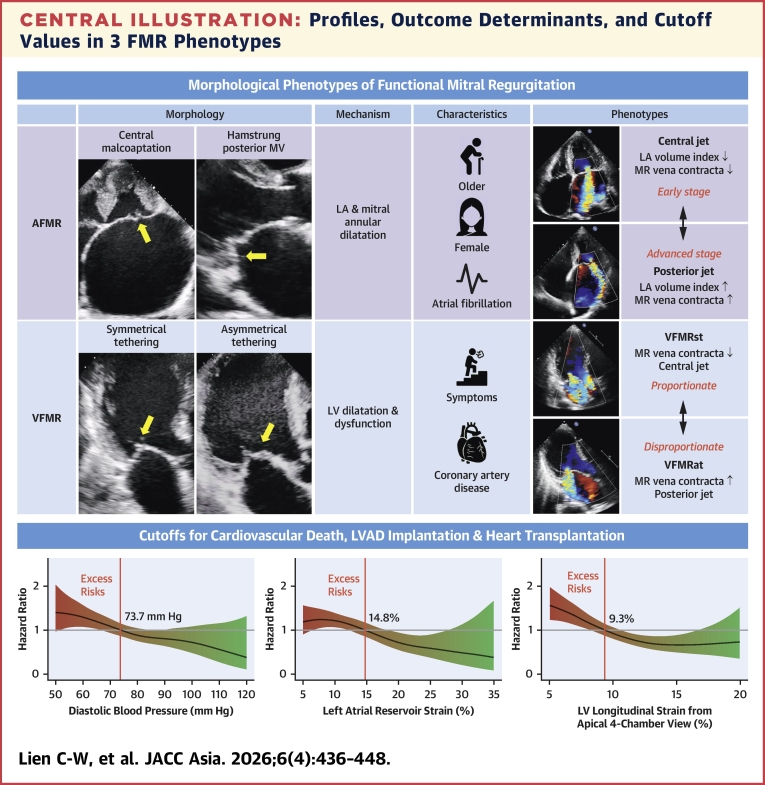
Profiles, Outcome Determinants, and Cutoff Values in 3 FMR Phenotypes Patients with atrial functional mitral regurgitation (AFMR) were older, predominantly female, and with higher prevalence of atrial fibrillation. Two types of AFMR were mitral valve (MV) central malcoaptation with central jet and hamstrung posterior MV with posterior jet. The latter displayed larger left atrial (LA) volume index and mitral regurgitation (MR) vena contracta, suggesting a more advanced stage. Two types of ventricular functional mitral regurgitation (VFMR) were ventricular functional mitral regurgitation with symmetrical tethering (VFMRat) and ventricular functional mitral regurgitation with symmetrical tethering (VFMRst). The latter had larger MR vena contracta, mimicking disproportionate MR. Adjusted spline curves indicated increased risk of the primary endpoint when diastolic blood pressure ≤73.7 mm Hg, left atrial reservoir strain (LASr) ≤14.8%, and left ventricular (LV) longitudinal strain from the apical 4-chamber view ≤9.3%. Yellow arrows indicate the site of malcoaptation or tethering. LVAD = left ventricular assist device.

### Measurement of LV and LA strain

A vendor-independent fully automated software (AutoStrain LV and LA Analysis, LOT 31.0; TomTec Imaging Systems) was used for strain analysis by an experienced imager (L.-T.Y.). Due to image quality limitations for apical windows in some patients (LV longitudinal strain was available from apical 2- and 3-chamber views in 554 and 427 patients, respectively), left ventricular longitudinal strain from the apical 4-chamber view (A4C-LVLS) was used for final analysis. For LA strain analysis, the reference point was end-diastole (R-R gating). The endocardial border tracking was modified if deemed inappropriate. Postadjustment values were used for analysis (semi-automated strain).^14^ Detailed methodology is presented in the Supplemental Methods. All measurements were performed blinded to all clinical and outcome information.

### Outcomes

Our primary composite endpoint was cardiovascular death (CVD) and equivalent of death (eg, LVAD implantation or HTx) at 5-year. Secondary endpoints were all-cause death (ACD) and equivalent of death (eg, LVAD implantation or HTx) at 5 years. Observation time was between the date of baseline TTE and death, LVAD implantation, HTx, or last follow-up (halted after 5 years). Mitral valve surgery (MVS) (repair or replacement) with or without concomitant CABG, isolated CABG without MVS, and extracorporeal membrane oxygenation implantation were treated as time-dependent covariates (eg, time-dependent procedures) for adjustment. As our institutes offer top-tier cardiovascular surgical techniques in Taiwan,^15^ it is unlikely that patients underwent the aforementioned procedures elsewhere. However, because heart failure (HF) hospitalizations could have occurred at other institutions, HF hospitalization was not included as an endpoint. Determinants of CVD and equivalent of death at 5 years for AFMR, VFMRat, and VFMRst were also analyzed. Mortality status, death date, and cause of death were retrieved from medical records and the National Health Insurance research database, which covers 99.5% of the Taiwanese population as a government-run, single-payer insurance plan.

### Statistical analysis

Continuous variables were expressed as mean ± SD or median (Q1-Q3), depending on their distribution. Normality was evaluated using the Shapiro-Wilk test. As most variables were non-normally distributed, nonparametric tests (Mann-Whitney *U* and Kruskal-Wallis) were used for between-group comparisons. Categorical data, presented as count and percentages, were compared using the chi-square test. Primary and secondary endpoints were analyzed using the Cox proportional hazards model, in which variables with clinical and pathophysiological relevance plus univariate *P* value <0.05 were chosen for multivariable analyses. LVEF, left ventricular end-systolic dimension index (LVESDi), left ventricular end-systolic volume index (LVESVi), and A4C-LVLS were placed in separate multivariable models to avoid collinearity (Pearson correlation coefficient *r* for LVEF and LVESDi, LVEF and LVESVi, LVEF, and LVESDi: −0.71, −0.81, and 0.66, respectively; *P* < 0.001). Event-free survival was estimated using the Kaplan-Meier method and compared using the log-rank statistic. Penalized smoothing splines were used to illustrate the risk of adverse events over the range of diastolic blood pressure (DBP), LASr, and A4C-LVLS compared with the cohort. All statistical analyses were performed using commercially available software (JMP 17 [SAS Institute] and R version 4.1.2 [R Foundation for Statistical Computing]). A 2-sided *P* value <0.05 was considered statistically significant.

## Results

### Baseline characteristics

The final cohort included 699 patients with ≥moderately severe FMR (median age 70 years) (Table 1, Supplemental Table 1): 151 (22%) with AFMR, 376 (54%) with VFMRst, and 172 (24%) with VFMRat. Compared with the AFMR subgroup, patients with VFMR were younger, were predominantly male, exhibited higher comorbidity burden, were more symptomatic, and used more antiplatelet agents and statins but fewer anticoagulants (all *P* ≤ 0.035). Patients with VFMR also had significantly larger LV dimensions/volumes, higher E/e′, lower LVEF, lower A4C-LVLS, impaired RV function, smaller mitral annulus index, smaller left atrial volume index (LAVi), less severe tricuspid regurgitation, and fewer MVS (all *P* ≤ 0.0001) (Table 1, Supplemental Table 1); LASr was similar between groups (*P* = 0.370). Compared with the VFMRst subgroup (Table 1), those with VFMRat had more atrial fibrillation; larger A4C-LVLS and LVEF (reflected by smaller LVESVi), as well as larger mitral regurgitation vena contracta (MR-VC); and LAVi (all *P* < 0.05). As expected, patients with VFMRat had less global LV hypokinesis compared with patients with VFMRst (*P* < 0.001).

**Table 1:** Baseline Characteristics in 3 Phenotypes of FMR

	Total	AFMR	VFMRst	VFMRat	*P* Value
	(N = 699,100%)	(n = 151,22%)	(n = 376,54%)	(n = 172,24%)	
Age (n = 699), y	70 (60-79)	74 (66-82)	69 (60-78)	66 (57-78)	<0.001
Female (n = 699)	320 (46)	98 (65)	150 (40)	71 (42)	<0.001
BSA (n = 699), m^2^	1.62 (1.51-1.75)	1.57 (1.47-1.70)	1.63 (1.51-1.75)	1.65 (1.54-1.78)	0.003
SBP (n = 667), mm Hg	123 (109-138)	126 (112-143)	122 (107-136)	122 (106-140)	0.101
DBP (n = 665), mm Hg	73 (65-84)	74 (66-84)	71 (64-82)	74 (64-88)	0.076
Hypertension (n = 690)	422 (61)	80 (56)	240 (64)	102 (60)	0.189
Hyperlipidemia (n = 690)	217 (31)	35 (24)	119 (32)	63 (37)	0.054
Diabetes mellitus (n = 692)	212 (31)	23 (16)	135 (36)	55 (32)	<0.001
Myocardial infarction (n = 690)	154 (22)	9 (6)	96 (26)	49 (29)	<0.001
CCI (n = 699)	3 (2-4)	2 (1-4)	3 (2-5)	3 (2-4)	<0.001
NYHA functional class ≥II (n = 691)	611 (89)	113 (76)	341 (92)	157 (92)	<0.001
Heart failure (n = 689)	523 (76)	92 (62)	297 (79)	134 (80)	<0.001
Prior CABG or PCI (n = 699)	250 (36)	21 (14)	162 (43)	67 (39)	<0.001
CAD (n = 699)	282 (40)	27 (18)	177 (47)	78 (45)	<0.001
AF at echocardiography (n = 699)	211 (30)	109 (72)	60 (16)	42 (24)^a^	<0.001
Antiplatelet (n = 695)	338 (49)	39 (26)	208 (55)	91 (54)	<0.001
Anticoagulant (n = 695)	153 (22)	57 (38)	60 (16)	36 (21)	<0.001
RAS inhibitor or ARNI (n = 697)	317 (45)	61 (41)	166 (44)	90 (53)	0.074
Beta-blocker (n = 695)	370 (53)	83 (55)	191 (51)	96 (57)	0.362
CCB (n = 695)	167 (24)	39 (26)	94 (25)	34 (20)	0.371
MRA (n = 695)	261 (38)	52 (35)	144 (38)	65 (38)	0.709
Statin (n = 695)	204 (29)	27 (18)	121 (32)	56 (33)	0.002
Diuretics (n = 695)	468 (67)	92 (61)	255 (68)	120 (72)	0.145
Digoxin (n = 695)	164 (24)	43 (29)	84 (22)	37 (22)	0.265
Echo parameters					
LVEF (n = 698), %	38 (30-48)	55 (50-60)	33 (28-39)	38 (30-46)^b^	<0.001
A4C-LVLS (n = 699), %	9.5 (7.2-12.5)	12.7 (9.5-15.8)	8.4 (6.4-10.5)	10.0 (7.6-12.1)^b^	<0.001
LVEDD (n = 698), mm	60 (55-66)	54 (50-59)	61 (56-66)	61 (57-68)	<0.001
LVESD (n = 698), mm	48 (41-55)	37 (33-42)	51 (45-56)	50 (44-58)	<0.001
LVESDi (n = 698), mm/m^2^	30 (25-34)	24 (20-26)	32 (28-35)	30 (27-34)^a^	<0.001
LVESVi (n = 698), mL/m^2^	61 (40-82)	28 (23-39)	70 (56-90)	63 (45-89)^c^	<0.001
LVEDVi (n = 698), mL/m^2^	96 (77-122)	63 (54-81)	106 (88-132)	100 (83-128)^a^	<0.001
LAVi (n = 666), mL/m^2^	63 (51-80)	85 (66-112)	58 (49-70)	62 (53-75)^c^	<0.001
LASr (n = 699), %	12.3 (9.1-16.1)	11.8 (9.1-14.6)	12.6 (9.2-17.0)	12.4 (8.7-15.8)	0.528
MR-VC (n = 697), mm	7.4 (6.7-8.4)	7.3 (6.5-8.2)	7.3 (6.7-8.2)	7.7 (7.0-8.7)^b^	0.001
MR-EROA (n = 144), cm^2^	0.31 (0.26-0.39)	0.34 (0.26-0.39)	0.32 (0.27-0.40)	0.30 (0.25-0.35)	0.340
MR jet					
Central	466 (67)	91 (60)	360 (96)	15 (9)	<0.001
Posterior	232 (33)	60 (40)	15 (4)	157 (91)	
TRPG (n = 683), mm Hg	39 (31-51)	39 (32-52)	40 (32-49)	38 (29-51)	0.856
TR ≥moderate-severe (n = 699)	147 (21)	53 (35)	61 (16)	33 (19)	<0.001
E/e′ (n = 535)	21 (17-29)	17 (14-23)	24 (19-31)	21 (18-27)^a^	<0.001
Mitral annulus (n = 698), mm	35.0 (32.0-37.0)	37.0 (34.0-40.0)	34.0 (32.0-36.0)	34.0 (32.0-36.0)	<0.001
Mitral annulus index (n = 698), mm/m^2^	21.4 (19.4-23.4)	24.1 (21.2-26.4)	21.2 (19.3-22.7)	20.4 (18.9-22.2)	<0.001
RV-FAC (n = 698), %	35.6 (30.2-41.2)	40.3 (35.6-45.9)	33.7 (28.5-39.3)	34.8 (29.0-39.9)	<0.001
RV function (n = 696)^d^	1 (0-2)	1 (0-1)	1 (1-2)	1 (0-2)	<0.001
RWMA (n = 699)					
None	251 (36)	146 (97)	85 (23)	20 (12)^b^	<0.001
Inferolateral wall	141 (20)	0 (0)	58 (15)	83 (48)	
Global hypokinesis	307 (44)	5 (3)	233 (62)	69 (40)	
Surgery					
Mitral valve repair or replacement at follow-up (n = 699)	84 (12)	32 (21)	31 (8)	21 (12)	<0.001

Values are n (%), mean ± SD, or median (Q1-Q3). *P* represents the comparison among the 3 groups. For comparison between VFMRst and VFMRat, there were significant differences between VFMRst and VFMRat groups.

A4C-LVLS = left ventricular longitudinal strain from the apical 4-chamber view; AF = atrial fibrillation; ARNI = angiotensin receptor neprilysin inhibitor; BSA = body surface area; CABG = coronary artery bypass grafting; CAD = coronary artery disease; CCB = calcium channel blocker; CCI = Charlson Comorbidity Index; DBP = diastolic blood pressure; E/e′ = peak mitral inflow velocity to early diastolic mitral annular velocity ratio; LASr = left atrial reservoir strain; LAVi = left atrial volume index; LV = left ventricular; LVEDD = left ventricular end-diastolic dimension; LVEDVi = left ventricular end-diastolic volume index; LVEDV = left ventricular end-diastolic volume; LVEF = left ventricular ejection fraction; LVESD = left ventricular end-systolic dimension; LVESDi = left ventricular end-systolic dimension index; LVESV = left ventricular end-systolic volume; LVESVi = left ventricular end-systolic volume index; MR = mitral regurgitation; MR-EROA = mitral regurgitation effective regurgitant orifice area; MR-VC = mitral regurgitation vena contracta; MRA = mineralocorticoid receptor antagonist; RAS = renin-angiotensin system; RV = right ventricular; RV-FAC = right ventricular fractional area change; RWMA = regional wall motion abnormality; SBP = systolic blood pressure; TR = tricuspid regurgitation; TRPG = tricuspid regurgitation peak gradient.

a 0.01 ≤ *P* < 0.05.

b *P* < 0.001.

c 0.001 ≤ *P* < 0.01.

d RV function represented as continuous variable from 0 to 4 (0 = normal; 1 = mildly reduced; 2 = moderately reduced; 3 = moderately to severely reduced; 4 = severely reduced).

In the AFMR subset (n = 151 [22%]), 91 (60%) patients had central jet and 60 (40%) had posterior jet (Table 2). Compared with patients with central MR, patients with posterior MR were predominantly male and had larger LVEF, A4C-LVLS, LVEDVi, LAVi, and MR-VC (all *P* ≤ 0.012) (Table 2).

**Table 2:** Baseline Characteristics in 2 Phenotypes of AFMR

	Central	Posterior	*P* Value
	(n = 91,60%)	(n = 60,40%)	
Age (n = 151), y	76 (67-82)	73 (63-82)	0.227
Female (n = 151)	67 (74)	31 (52)	0.005
BSA (n = 151), m^2^	1.53 (1.45-1.70)	1.60 (1.51-1.72)	0.098
SBP (n = 143), mm Hg	128 (112-150)	123 (113-131)	0.052
DBP (n = 142), mm Hg	76 (68-90)	72 (66-80)	0.053
Hypertension (n = 144)	54 (62)	26 (45)	0.052
Hyperlipidemia (n = 144)	24 (28)	11 (19)	0.252
Diabetes mellitus (n = 145)	16 (18)	7 (12)	0.335
Myocardial infarction (n = 144)	5 (6)	4 (7)	0.759
CCI (n = 151)	2 (1-4)	2 (1-3)	0.345
NYHA functional class ≥II (n = 148)	68 (77)	45 (75)	0.749
Heart failure (n = 148)	52 (59)	40 (66)	0.349
Prior CABG or PCI (n = 151)	15 (16)	6 (10)	0.251
CAD (n = 151)	18 (19)	9 (15)	0.449
AF at echocardiography (n = 151)	62 (68)	47 (78)	0.166
Antiplatelet (n = 150)	28 (31)	11 (18)	0.075
Anticoagulant (n = 150)	37 (41)	20 (33)	0.334
RAS inhibitor or ARNI (n = 150)	36 (40)	25 (42)	0.838
Beta-blocker (n = 150)	52 (58)	31 (52)	0.461
CCB (n = 150)	27 (30)	12 (20)	0.166
MRA (n = 150)	29 (32)	23 (38)	0.442
Statin (n = 150)	18 (20)	9 (15)	0.430
Diuretics (n = 150)	59 (66)	33 (55)	0.194
Digoxin (n = 150)	25 (28)	18 (30)	0.768
Echo parameters			
LVEF (n = 151), %	53 (48-58)	58 (53-61)	0.012
A4C-LVLS (n = 151)	12.1 (8.7-14.2)	13.9 (12.0-16.6)	<0.001
LVEDD (n = 151), mm	52 (49-58)	56 (51-63)	0.011
LVESD (n = 151), mm	36 (33-41)	39 (32-44)	0.213
LVESDi (n = 151), mm/m^2^	24 (21-25)	24 (19-27)	0.777
LVESVi (n = 151), mL/m^2^	28 (23-37)	31 (24-40)	0.183
LVEDVi (n = 151), mL/m^2^	59 (51-73)	74 (56-87)	0.003
LAVi (n = 143), mL/m^2^	78 (63-97)	96 (77-145)	<0.001
LASr (n = 151), %	11.3 (9.1-14.2)	12.1 (9.3-17.9)	0.193
MR-VC (n = 151), mm	7.0 (6.4-8.2)	7.5 (6.8-8.6)	0.010
TRPG (n = 149), mm Hg	41 (33-53)	38 (30-47)	0.099
TR ≥ moderate-severe (n = 151)	34 (37)	19 (32)	0.471
E/e′ (n = 98)	18 (14-23)	17 (13-21)	0.716
Mitral annulus (n = 151), mm	36.0 (34.0-39.0)	38.0 (35.0-41.0)	0.011
Mitral annulus index (n = 151), mm/m^2^	24.1 (21.1-26.1)	24.3 (21.2-26.6)	0.505
RV-FAC (n = 151), %	40.3 (35.3-45.9)	40.5 (36.1-45.9)	0.824
RV function (n = 151)	1 (0-1)	0 (0-1)	0.818
Surgery			
Mitral valve repair or replacement (n = 151)	18 (20)	16 (27)	0.324

Values are n (%) or median (Q1-Q3).

AFMR = atrial functional mitral regurgitation; PCI = percutaneous coronary intervention; other abbreviations as in Table 1.

### Surgical procedures

Before observation ended (death, LVAD implantation, HTx, or 5-year follow-up), 84 patients received MVS at an average of 3.7 ± 6.8 months from baseline, including 53 (63%) MV repair (M-TEER in 3) and 31 (37%) MV replacement (bioprosthesis in 28 and mechanical valve in 3); 25 (30%) patients received concomitant CABG, 20 (24%) received concomitant maze procedure, and 34 (40%) received concomitant tricuspid annuloplasty at the time of MVS. Thirteen patients had isolated CABG without MVS during observation at an average of 6 ± 14 months from baseline. At an average of 8.8 ± 7.8 months from baseline, 29 (4%) patients had LVAD or HTx.

### Determinants for primary composite endpoints

All follow-ups were 100% completed by December 31, 2022. Median follow-up for the primary endpoints was 2.6 years (Q1-Q3: 0.9-5 years), and 193 patients reached the primary endpoint (29 patients received LVAD/HTx, 164 had CVD). Univariable determinants for the primary endpoint are summarized in Supplemental Table 2. VFMRst and VFMRat were univariably associated with the primary endpoint (*P* ≤ 0.030). Separate multivariable models (Table 3) found male sex, lower DBP, reduced right ventricular fractional area change (RV-FAC), LVEF, and A4C-LVLS (cutoff: 9.3%), lower LASr, as well as larger LVESDi, and LVESVi were independently linked to the primary endpoint (all *P* ≤ 0.043); however, MR phenotypes were not associated with the primary endpoints after adjustments.

**Table 3:** Multivariable Determinants of CVD/HTx/LVAD (N = 193) at 5-Year Follow-Up

	LVEF Model		LVESDi Model		LVESVi Model		A4C-LVLS model	
	HR (95% CI)	*P* Value	HR (95% CI)	*P* Value	HR (95% CI)	*P* Value	HR (95% CI)	*P* Value
MR phenotype (Ref: AFMR)								
VFMRst	0.76 (0.43-1.36)	0.354	0.89 (0.54-1.48)	0.661	0.84 (0.51-1.36)	0.476	1.02 (0.64-1.62)	0.928
VFMRat	0.93 (0.52-1.64)	0.794	1.07 (0.64-1.81)	0.789	0.97 (0.58-1.63)	0.915	1.22 (0.74-1.99)	0.433
Female	0.73 (0.53-1.02)	0.066	0.66 (0.47-0.92)	0.013	0.74 (0.54-1.03)	0.077	0.69 (0.50-0.96)	0.025
NYHA functional class ≥II	1.96 (0.90-4.27)	0.091	1.68 (0.81-3.50)	0.166	1.79 (0.86-3.72)	0.120	1.91 (0.92-3.95)	0.083
DBP, per 5 mm Hg	0.93 (0.88-0.98)	0.005	0.93 (0.88-0.98)	0.009	0.94 (0.89-0.99)	0.018	0.92 (0.88-0.97)	0.003
RV-FAC	0.99 (0.97-1.01)	0.279	0.98 (0.96-1.00)	0.060	0.98 (0.96-1.00)	0.117	0.98 (0.96-1.00)	0.038
LASr	0.97 (0.94-1.00)	0.024	0.96 (0.93-0.99)	0.007	0.96 (0.94-0.99)	0.008	0.97 (0.94-1.00)	0.035
LVEF, per 10%	0.75 (0.61-0.91)	0.004	—	—	—	—	—	—
LVESDi, per mm/m^2^	—	—	1.03 (1.01-1.06)	0.018	—	—	—	—
LVESVi, per 10 mL/m^2^	—	—	—	—	1.09 (1.04-1.15)	<0.001	—	—
A4C-LVLS >9.3%	—	—	—	—	—	—	0.70 (0.49-0.99)	0.043
Time-dependent procedures^a^	1.62 (1.07-2.44)	0.023	1.40 (0.93-2.10)	0.110	1.50 (1.00-2.26)	0.052	1.32 (0.88-1.98)	0.173

Models also adjusted for stratified age (cutoff at 70 years), MR-VC, statin, beta-blocker, antiplatelet, CCB, MRA, and diuretics.

CVD = cardiovascular death; HTx = heart transplantation; LVAD = left ventricular assist device; VFMRat = ventricular functional mitral regurgitation with asymmetrical tethering; VFMRst = ventricular functional mitral regurgitation with symmetrical tethering; other abbreviations as in Table 1.

a Mitral valve surgery (repair or replacement) or isolated CABG.

**Figure 1:**
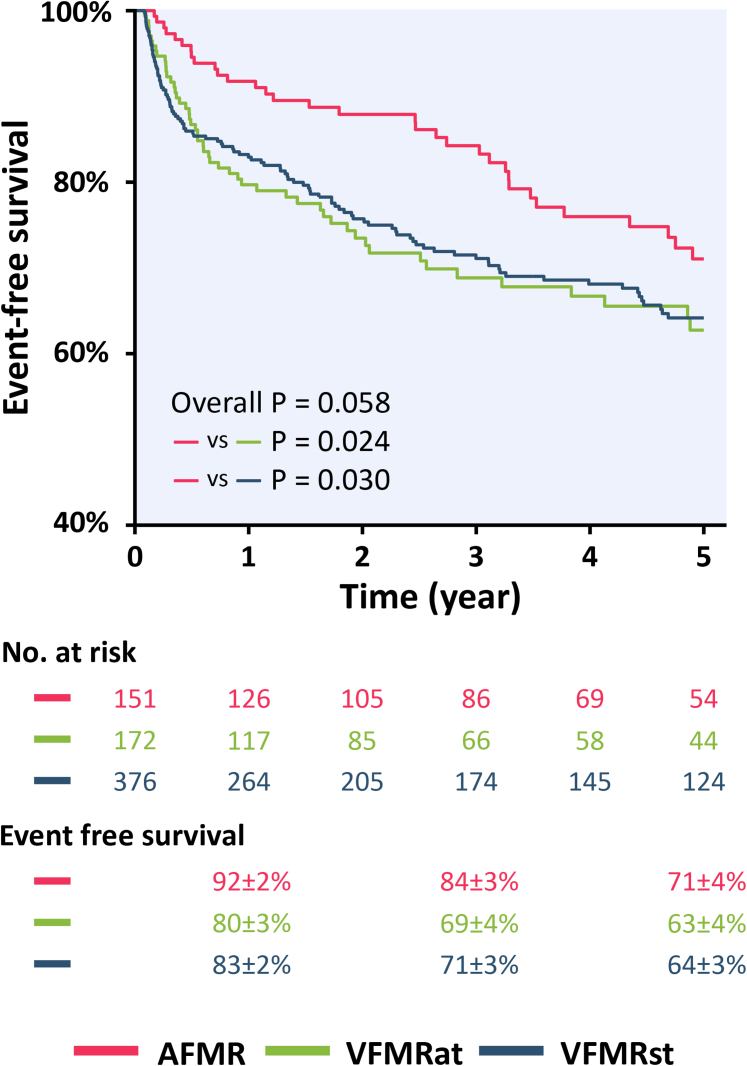
Kaplan-Meier Curves for Primary Endpoints in 3 FMR Phenotypes Kaplan-Meier curves show event-free survival over a 5-year follow-up period among patients with atrial functional mitral regurgitation (AFMR) (pink), ventricular functional mitral regurgitation with asymmetrical tethering (VFMRat) (green), and ventricular functional mitral regurgitation with symmetrical tethering (VFMRst) (blue). Although the overall comparison among the 3 functional mitral regurgitation phenotypes showed a borderline difference (*P* = 0.058), patients with AFMR had significantly better survival than those with either VFMRat (*P* = 0.024) or VFMRst (*P* = 0.030), whereas survival did not differ between the two VFMR groups. Numbers at risk and 1-, 3-, and 5-year event-free survival rates (mean ± SE) are provided below the graph.

The non-adjusted Kaplan-Meier curve found that the 5-year event-free survival for AFMR, VFMRat, and VFMRst was 71% ± 4%, 63% ± 4%, and 64% ± 3%, respectively (*P* = 0.030 for AFMR and VFMRst; *P* = 0.024 for AFMR and VFMRat) (Figure 1). AFMR patients with posterior jet had similar 5-year event-free survival (HR: 1.16; 95% CI: 0.57-2.33; *P* = 0.678) as compared with those with central jet.

### Incremental analysis for the primary endpoint

Incremental analysis was performed by stepwise addition of DBP, LV parameters (LVEF, LVESDi, LVESVi, A4C-LVLS), and LASr to the core model (MR phenotypes, stratified age, sex, and NYHA functional class ≥II). Adding DBP to the core model provided additional prognostic value (*P* ≤ 0.001). Inclusion of all 4 LV parameters further improved prognostic value (*P* < 0.001), and subsequent addition of LASr offered additional value (*P* ≤ 0.007) (Supplemental Figure 2).

### Determinants for secondary endpoints of ACD, HTx, and LVAD

At a median follow-up of 2.6 years (Q1-Q3: 0.9-5 years), 356 (51%) patients reached the secondary endpoints (29 patients received LVAD/HTx, 327 had ACD). Results of univariable analysis are shown in Supplemental Table 3; VFMRst was univariably associated with secondary endpoints (*P* = 0.003). Separate multivariable models (Supplemental Table 4) found that higher Charlson Comorbidity Index and lower DBP, RV-FAC, and LASr were independently associated with secondary endpoints (all *P* ≤ 0.049); MR phenotypes were not associated with secondary endpoints after adjustment.

### Cutoffs for primary/secondary endpoints via age- and sex-adjusted spline curves

**Figure 2:**
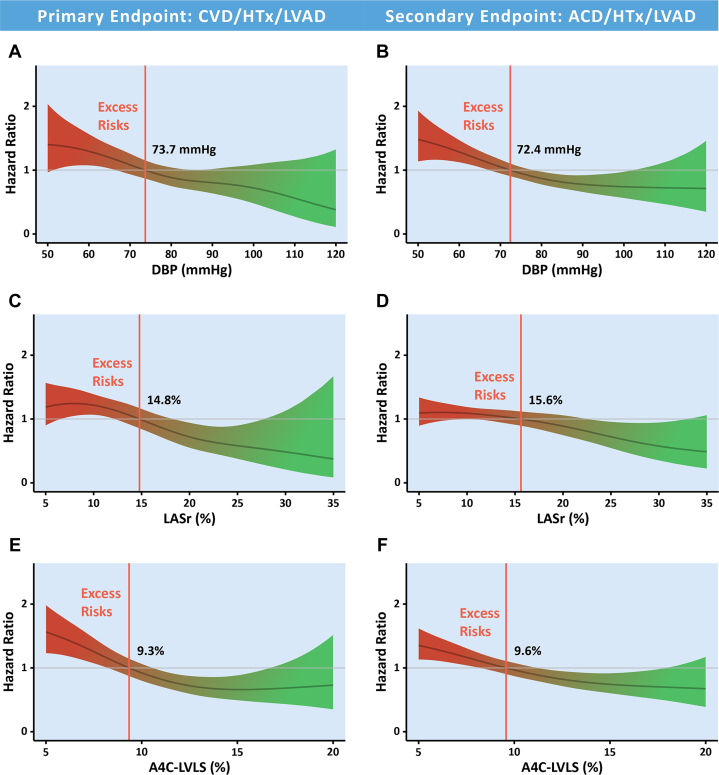
Adjusted Splines for Primary and Secondary Endpoints The HR = 1 line indicates the cohort's average mortality, with excess risk present at values >1. Spline curves adjusted for stratified age (cutoff at 70 years), sex, right ventricular fractional area change (RV-FAC), and procedures revealed increased risk of the primary endpoint (cardiovascular death [CVD]/heart transplantation [HTx]/left ventricular assist device [LVAD]) and secondary endpoint (all-cause death [ACD]/HTx/LVAD) when the diastolic blood pressure (DBP) was (A) <73.7 mm Hg and (B) <72.4 mm Hg, respectively. (C, D) Increased risk of primary and secondary endpoints when left atrial reservoir strain (LASr) was <14.8% and <15.6%, respectively. Increased risk of primary and secondary endpoints when left ventricular longitudinal strain from the apical 4-chamber view (A4C-LVLS) was (E) <9.3% and (F) <9.6%, respectively. Red-shaded areas indicate excess risk, while green-shaded areas represent protective ranges associated with lower risk. Vertical dashed lines mark risk inflection thresholds.

Overall, the risks for primary and secondary endpoints gradually increased when DBP fell below 73.7 mm Hg and 72.4 mm Hg, respectively; the corresponding LASr cutoffs for primary and secondary endpoints were 14.8% and 15.6%, respectively, and the corresponding A4C-LVLS cutoffs for the primary and secondary endpoints were 9.3% and 9.6%, respectively (Figure 2). A4C-LVLS >9.3% and larger LVEF were both associated with better outcomes in a head-to-head comparison model (Supplemental Table 5).

### Determinants of outcomes in 3 FMR subgroups

Univariable analysis for the 3 FMR-phenotypes are shown in Supplemental Table 6. Determinants of the primary endpoint in multivariable models were: 1) larger MR-VC, larger LAVi, and impaired RV function for AFMR (all *P* ≤ 0.041); 2) atrial fibrillation, lower LVEF, lower LASr, lower A4C-LVLS, and larger LVESVi for VFMRat (all *P* ≤ 0.045); and 3) lower DBP, lower LVEF, lower A4C-LVLS, lower LASr, and larger LVESVi for VFMRst (all *P* ≤ 0.041) (Supplemental Table 7).

## Discussion

We report for the first time using comprehensive FMR phenotypical classifications and correlations with outcome determinants. Our principal findings are the following. First, of the 3 phenotypes, VFMRst was the most prevalent (54%) and AFMR was the least prevalent (22%). The prevalence of AFMR with hamstrung PML (40%) was higher than previously reported.^16^ Second, although AFMR was classified morphologically, the median LVEF was >50%, median LVEDVi ≤75 mL/m^2^ and LAVi ≥40 mL/m^2^, matching previously proposed criteria.^17^ Third, besides conventional indexes, lower RV-FAC, DBP, LVEF, A4C-LVLS, LASr, and larger LVESDi and LVESVi were independent determinants of the primary endpoint. Fourth, AFMR had better 5-year crude event-free survival compared with VFMRst and VFMRat, likely due to better cardiac function; however, MR phenotypes were not associated with the primary endpoints after adjustment for covariates. Fifth, DBP, LASr, and A4C-LVLS cutoffs for excess risks of the primary/secondary endpoints were 72 to 74 mm Hg, 14.8% to 15.6%, and 9.3% to 9.6%, respectively. Sixth, LV indexes (LVEF, LVESVi, A4C-LVLS) were determinants for the VFMR subgroup while LAVi, RV function, and MR-VC were determinants for the AFMR subgroup.

### Morphological phenotypes of FMR

Conflicting results from landmark trials highlighted the importance of revisiting VFMR classification.^2^^,^^3^^,^^8^ Based on MV morphology,^9^ we proposed 2 VFMR subtypes: VFMRat and VFMRst. AFMR, on the other hand, has previously been classified using fixed parameters, including LVEF cutoffs, LV dimensions/volumes, or absence of regional WMA.^13^^,^^16^^,^^17^ However, in clinical practice, these patients may present with mildly dilated LV or slightly reduced LVEF due to MR-related volume overload or atrial fibrillation–induced cardiomyopathy,^18^ making the 50% LVEF cutoff potentially unreliable. Classifying FMR phenotypes based on MV morphology, as proposed in this study, may offer a more comprehensive understanding of the underlying mechanisms and better capture the full spectrum of AFMR.^19^

### AFMR: a heterogeneous entity

AFMR, which typically occurs with atrial fibrillation and/or HF with preserved ejection fraction, has drawn more attention recently owing to emerging transcatheter therapeutics.^20^ Although AFMR was classified via unique mitral morphology rather than LV cutoffs herein, our AFMR patients exhibited relatively preserved LVEF, with mean LVEDVi and LAVi aligning with previous AFMR studies.^16^^,^^21^ Additionally, our AFMR patients were less symptomatic than VFMR patients, which may explain why AFMR patients tended to be overlooked and present at late stage. Albeit unsatisfactory, our study revealed that AFMR had better crude survival than VFMR owing to better cardiac function, which was not necessarily associated with the phenotype per se (Table 3). Larger MR-VC, LAVi, and reduced RV function, which linked to adverse outcomes (Supplemental Table 7), may help identify high-risk AFMR patients requiring aggressive MV intervention, including M-TEER.^21^

In patients with AFMR, significant LA enlargement can displace the posterior MV annulus beyond the crest of the myocardial LV inlet, causing atriogenic PML tethering (“hamstrung PML”) and a posterior MR jet—an uncommon subtype associated with a trend toward worse outcomes and lower M-TEER success rate.^13^^,^^20^^,^^22^ Mesi et al^16^ found posterior jet in 23% of patients with ≥moderate-severe AFMR; these patients had larger mitral annulus, more severe MR, and numerically higher number of deaths compared with central MR. Likewise, von Stein et al^22^ reported that AFMR with atriogenic hamstringing was associated with worse procedural and clinical outcomes after M-TEER compared with AFMR with central jet, although their study was limited by a small sample size. Our study showed a higher prevalence (40%) of the hamstrung PML subtype and that mitral annulus normalized by body surface area was similar between two subtypes, consistent with a recent study.^22^ Patients with the hamstrung PML subtype exhibited larger LAVi and MR-VC, aligning with the hypothesis by Farhan et al^20^ that hamstrung PML results from a more advanced disease course (Central Illustration). Whether this subtype is the final common pathway for all patients with AFMR or the consequence of different atrial remodeling still needs further investigation.

### Disproportionate and proportionate VFMR: from the viewpoint of MV morphology

The concept of disproportionate FMR, introduced by Grayburn et al^7^ to explain conflicting results between two landmark trials, is attributed to asymmetrical papillary muscle displacement and inferolateral WMA.^4^^,^^12^ Although limited by LV volume underestimation and challenged by newer volume-based models, this proportionateness hypothesis remains popular as clinicians seek explanations for discrepancies between landmark trials.^23^^,^^24^ Our phenotypical classification of FMR aligns with this hypothesis: compared with the VFMRst subgroup, the VFMRat subgroup resembled disproportionate FMR, with significantly smaller LV volumes and larger MR-VC (Central Illustration, Table 1). However, MR EROA was similar between the groups, possibly due to: 1) underestimation of proximal isovelocity surface area–derived EROA in eccentric jets;^25^ and 2) incomplete EROA data.

### New insights into low DBP and unfavorable outcomes in FMR

Low DBP is linked to adverse outcomes in various conditions, including HF with preserved ejection fraction,^26^ ischemic heart disease,^27^ and aortic regurgitation,^28^ due to subclinical myocardial damage from reduced coronary perfusion^29^ or aortic stiffness.^30^ Our study is the first to report the association between low DBP and adverse outcomes in FMR, with a cutoff (72.4-73.7mm Hg) similar to prior findings.^27^^,^^28^ Unlike other studies, we did not observe a J-curve phenomenon, likely because our FMR patients were frail and had relatively low DBP compared with hypertensive populations.

### The role of LV strain and LASr in FMR: the LV-LA interplay

While LVEF is crucial for interventional decisions in FMR,^5^^,^^6^ it cannot detect subclinical LV dysfunction. Few studies have explored LVGLS in FMR. Namazi et al^10^ reported increased mortality in VFMR patients with LVGLS <7.0%, and our study confirmed its prognostic value across the FMR cohort. We found disproportionately lower A4C-LVLS in AFMR patients (12.7% [Q1-Q3: 9.5%-15.8%]) despite relatively preserved LVEF, likely due to: 1) MR-associated reductions in LVGLS;^31^ 2) LVEF overestimation in significant MR, with a median LVEF of 55% (Q1-Q3: 50%-60%) suggesting systolic dysfunction; and 3) extremely enlarged LAVi (85 mL/m^2^), reflecting LV-LA interdependence.^32^ In short, significant LA enlargement may limit LV base descent, reducing LVLS, while LV base dynamics significantly influence LA reservoir function.^32^

LASr was similarly low across all groups (*P* = 0.528), even though AFMR patients had better LVEF than the VFMR subgroups. This may be explained by the presence of severely enlarged LA in all 3 groups (Table 1). Nonetheless, our study identified LASr, a marker of atrial fibrosis^11^ and predictor of poor outcomes,^33^^,^^34^ as a strong determinant of adverse events. This is likely because LASr reflects LV-LA interdependence, captures the function of both chambers, and allows early detection of elevated LV filling pressure and LA dysfunction.^35^ While one study in ≥ moderate VFMR demonstrated the incremental prognostic value of LASr over LAVi,^11^ we found its usefulness to be limited in AFMR patients with severely dilated left atria, indicating advanced atrial dysfunction (Supplemental Table 7).

With the rise of automated strain imaging, LV and LA strain have become easy to quantify and may serve as sensitive markers of early cardiac dysfunction. In our cohort, LASr demonstrated prognostic value; however, its role in guiding interventions remains uncertain and awaits validation in future trials.

### Clinical implications

This study offers several clinical insights. First, although AFMR patients showed better crude survival than those with VFMR, their 5-year event-free survival remained suboptimal and warrants attention. AFMR with a hamstrung PML may indicate a more advanced stage of disease. Second, comparable adjusted outcomes across MR phenotypes may be due to confounding baseline characteristics and limited adoption of contemporary interventions. However, this does not indicate equivalent M-TEER effectiveness, as only a small number (n = 3) underwent the procedure. Third, morphology-based AFMR definitions may better capture late-stage cases, as supported by recent studies^36^ using LVEF ≥40% rather than 50%. Fourth, beyond standard indices, DBP, A4C-LVLS, and LASr may aid in identifying high-risk patients for timely intervention. Fifth, although VFMRst, which mimics proportionate FMR, and VFMRat, resembling disproportionate MR, showed similar adjusted survival, their responses to M-TEER may differ. Evaluating this hypothesis in a TEER-treated cohort would be compelling, as it could uncover phenotype-specific therapeutic benefits and enhance the clinical applicability of the proposed classification.

### Study limitations

Being retrospective, the study was subject to potential selection bias. Besides, it is difficult to discern whether the outcome determinants found in our cohort were associated with MR severity or underlying myocardial disease. Also, it remains unclear whether theses determinants and their respective cutoffs represent a point of no return or reflect potential responsiveness to M-TEER. For example, Kaneko et al^37^ found that M-TEER was associated with better outcomes when the LAVi was <100 mL/m^2^. Additionally, LV longitudinal strain was derived solely from A4C views. However, Salaun et al^38^ reported that A4C-LVLS independently predicts mortality in AS, suggesting it may serve as an alternative to LVGLS for prognostic evaluation. MR EROA data were unavailable in some cases, but prior studies indicate that MR-VC is equally reliable for central and eccentric jets.^39^ Last, guideline-directed medical therapy was suboptimal, and only 2 patients underwent M-TEER due to lack of reimbursement by national health insurance, preventing us from assessing outcome improvement across phenotypes.

## Conclusions

The current FMR study supplements existing literature by providing morphological insights. AFMR had the best, albeit unsatisfactory, survival of the 3 phenotypes. AFMR with hamstrung PML is not uncommon and could be a form of advanced AFMR. VFMRat mimics disproportionate MR compared with VFMRst. Besides conventional RV and LV functional indices, lower DBP, LASr, and A4C-LVLS were independently associated with worse outcomes in FMR. Further studies are needed to verify the impact of phenotypical classifications on treatment strategies.

## Funding Support and Author Disclosures

This research was supported by the National Science and Technology Council (114-2314-B-002-220) and National Taiwan University Hospital (114-IF0006). The authors have reported that they have no relationships relevant to the contents of this paper to disclose.
