# Supplementary material for: Profiles of Comprehensive Morphological Phenotypes and Outcomes in Functional Mitral Regurgitation
Source: JACC Asia. 2026 Jan 21;6(4):436–48. doi: 10.1016/j.jacasi.2025.11.014 (PMC13080753; doi:10.1016/j.jacasi.2025.11.014)
Supplement: Supplemental Material [file mmc1.docx]

**Supplemental Materials**

|  | **Page** |
| --- | --- |
| Supplemental Figure 1. Study flow | 2 |
| Supplemental Methods | 3 |
| Supplemental Table 1. Baseline characteristics in 2 phenotypes of FMR | 4 |
| Supplemental Table 2. Univariable predictors of CVD/HTx/LVAD at 5-year follow-up | 5 |
| Supplemental Figure 2. Incremental prognostic value of adding LASr and left ventricular parameters to DBP for CVD/HTx/LVAD Prediction | 6 |
| Supplemental Table 3. Univariable predictors of ACD/HTx/LVAD (n=356) during 5-year follow-up | 7 |
| Supplemental Table 4. Multivariable predictors of ACD/HTx/LVAD (n=356) at 5-year follow-up | 8 |
| Supplemental Table 5. Head-to-head comparison of A4C-LVLS and LVEF for the endpoint of CVD/HTx/LVAD at 5-year follow-up | 9 |
| Supplemental Table 6. Univariable predictors of CVD/HTx/LVAD in 3 subgroups in 5-year follow-up | 10 |
| Supplemental Table 7. Multivariable predictors of CVD/HTx/LVAD in 3 subgroups in 5-year follow-up | 11 |

**Supplemental Figure 1. Study flow**

**
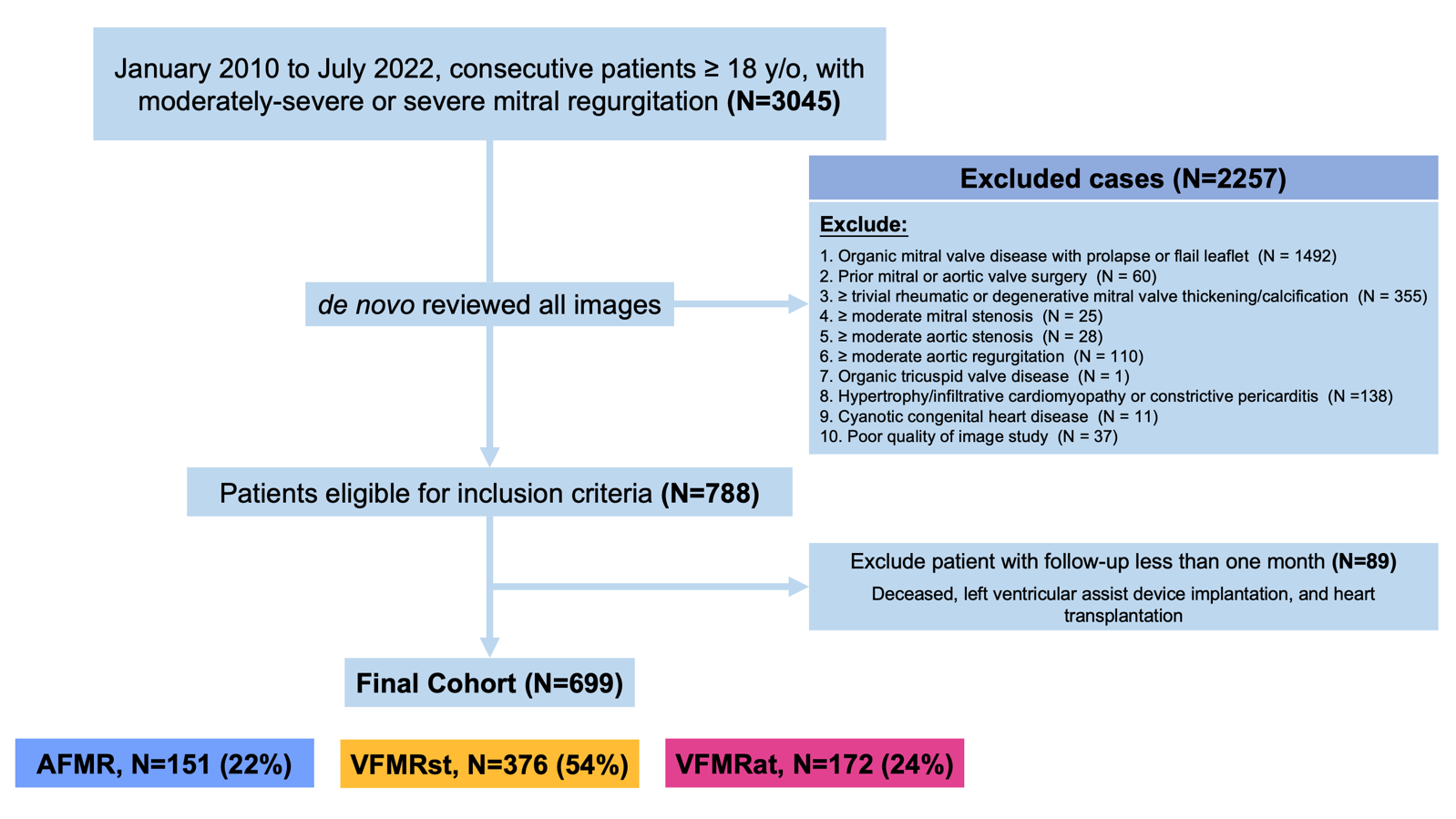
**

**Supplemental Methods:**

A vendor-independent fully-automated software(AutoStrain LV and LA Analysis, LOT 31.0; TomTec Imaging Systems, Unterschleissheim, Germany) was used for strain analysis by an experienced imager(LTY). Limited by the fact that in some patients, complete apical windows were not available(LV longitudinal strain was available from apical 2- and 3-chamber views in 554 and 427 patients, respectively), we used LV longitudinal strain derived from the A4C(A4C-LVLS) for final analysis. For A4C-LVLS analysis, the software used a knowledge-based AI algorithm to determine the endocardial border after selecting A4C, then performed speckle tracking analysis over a full cardiac cycle, averaging the end-systolic strain(identified at minimal LV-volume) from 6 segments; the absolute value of A4C-LVLS was used for analysis. Similarly, for LAS analysis, the software automatically tracks endocardial border of LA after A4C-view was selected; the reference point was end-diastole (R-R gating). LAS, averaged over 6 segments, consisted of 3 major LA functions: reservoir (LASr), conduit, and contractile strains. Due to high prevalence of AFib(30%) herein, only LASr was used for analysis. For both A4C-LVLS and LASr analysis, the accuracy of the endocardial border tracking was checked, and in 72% cases for A4C-LVLS and 57% cases for LASr, part of the endocardial tracking was modified if deemed inappropriate. The post-adjustment value was used for analysis(semi-automated strain).

**Supplemental Table 1. Baseline characteristics in 2 phenotypes of FMR**

|  | **Total**  **N=699** | **VFMR**  **N=548** | **AFMR**  **N=151** | P |
| --- | --- | --- | --- | --- |
| Age(n=699) | 70[60–79] | 69[59–78] | 74[66–82] | **<.001** |
| Female(n=699) | 320(46) | 222(41) | 98(65) | **<.001** |
| Body surface area, *m^2^*(n=699) | 1.62[1.51–1.75] | 1.63[1.51–1.75] | 1.57[1.47–1.70] | **.003** |
| Systolic blood pressure, mmHg(n=667) | 123[109–138] | 122[107–138] | 126[112–143] | **.034** |
| Diastolic blood pressure, mmHg(n=665) | 73[65–84] | 72[64–84] | 74[66–84] | .160 |
| Hypertension(n=690) | 422(61) | 342(63) | 80(56) | .122 |
| Hyperlipidemia(n=690) | 217(31) | 182(33) | 35(24) | **.035** |
| Diabetes mellitus(n=692) | 212(31) | 190(35) | 23(16) | **<.001** |
| Myocardial infarction(n=690) | 154(22) | 145(27) | 9(6) | **<.001** |
| CCI(n=699) | 3(2-4) | 3(2-4) | 2(1-4) | **<.001** |
| NYHA ≥ II(n=691) | 611(89) | 498(92) | 113(76) | **<.001** |
| Heart failure(n=689) | 523(76) | 431(80) | 92(62) | **<.001** |
| Prior CABG or PCI(n=699) | 250(36) | 229(42) | 21(14) | **<.001** |
| CAD(n=699) | 282(40) | 177(47) | 27(18) | **<.001** |
| AF at echo (n=699) | 211(30) | 102(19) | 109(72) | **<.001** |
| Antiplatelet(n=695) | 338(49) | 299(55) | 39(26) | **<.001** |
| Anticoagulant(n=695) | 153(22) | 96(18) | 57(38) | **<.001** |
| RASi or ARNi(n=697) | 317(45) | 256(47) | 61(41) | .180 |
| Beta-blocker(n=695) | 370(53) | 287(53) | 83(55) | .561 |
| Calcium channel blocker(n=695) | 167(24) | 128(23) | 39(26) | .526 |
| MRA(n=695) | 261(38) | 209(38) | 52(35) | .407 |
| Statin(n=695) | 204(29) | 177(32) | 27(18) | **<.001** |
| Diuretics(n=695) | 468(67) | 376(69) | 92(61) | .080 |
| Digoxin(n=695) | 164(24) | 121(22) | 43(29) | .104 |
| Nitrate(n=695) | 131(19) | 108(20) | 23(15) | .205 |
| Hydralazine(n=695) | 28(4) | 26(5) | 2(1) | .033 |
| **Echo parameters** |  |  |  |  |
| LVEF, %(n=698) | 38[30–48] | 35[28–41] | 55[50–60] | **<.001** |
| A4C-LS, %(n=699) | 9.5[7.2–12.5] | 8.7[6.7–11.1] | 12.7[9.5–15.8] | **<.001** |
| LVEDD, mm(n=698) | 60[55–66] | 61[56–67] | 54[50–59] | **<.001** |
| LVESD, mm(n=698) | 48[41–55] | 51[45–56] | 37[33–42] | **<.001** |
| LVESDi, mm/m^2^(n=698) | 30[25–34] | 31[27–35] | 24[20–26] | **<.001** |
| LVESVi, ml/m^2^(n=698) | 61[40–82] | 69[53–90] | 28[23–39] | **<.001** |
| LVEDVi, ml/m^2^(n=698) | 96[77–122] | 104[87–131] | 63[54–81] | **<.001** |
| LAVi, ml/m^2^(n=666) | 63[51–80] | 59[50–72] | 85[66–112] | **<.001** |
| LASr, %(n=699) | 12.3[9.1–16.1] | 12.4[9.1–16.6] | 11.8[9.1–14.6] | .370 |
| MR-VC, mm(n=697) | 7.4[6.7–8.4] | 7.4[6.8–8.4] | 7.3[6.5–8.2] | .140 |
| MR EROA, cm^2^(n=144) | 0.31[0.26–0.39] | 0.31[0.26–0.39] | 0.34[0.26–0.39] | .551 |
| MR jet(n=698): central | 466(67) | 375(69) | 91(60) | .058 |
| posterior | 232(33) | 172(31) | 60(40) |  |
| TRPG, mmHg(n=683) | 39[31–51] | 40[31–50] | 39[32–52] | .790 |
| TR ≥ moderate-severe(n=699) | 147(21) | 94(17) | 53(35) | **<.001** |
| E/e’ (n=535) | 21[17–29] | 23[18–30] | 17[14–23] | **<.001** |
| Mitral annulus, mm(n=698) | 35.0[32.0–37.0] | 34.0[32.0–36.0] | 37.0[34.0–40.0] | **<.001** |
| Mitral annulus index, mm/m^2^(n=698) | 21.4[19.4–23.4] | 21.0[19.1–22.6] | 24.1[21.2–26.4] | **<.001** |
| RV-FAC, %(n=698) | 35.6[30.2–41.2] | 34.1[28.6–39.4] | 40.3[35.6–45.9] | **<.001** |
| RV function(n=696) | 1[0–2] | 1[1–2] | 1[0–1] | **<.001** |
| RWMA(n=699), none | 251(36) | 105(19) | 146(97) | **<.001** |
| inferolateral wall | 141(20) | 141(26) | 0(0) |  |
| global hypokinesis | 307(44) | 302(55) | 5(3) |  |
| **Surgery** |  |  |  |  |
| Mitral valve repair or replacement(n=699) | 87(12) | 53(10) | 34(23) | **<.001** |

See Table 1 for abbreviations and definitions

**Supplemental Table 2. Univariable** **determinants of CVD/HTx/LVAD (n=193) at 5-year follow-up**

|  | **HR (95% CI)** | P |
| --- | --- | --- |
| Age per year | 1.01(0.99-1.02) | .050 |
| CCI | 1.05(0.98-1.12) | .100 |
| Female | 0.59(0.44-0.79) | **.004** |
| SBP | 0.99(0.98-0.99) | **.004** |
| DBP | 0.98(0.97-0.99) | **.0002** |
| NYHA ≥ II | 2.82(1.49-5.33) | **.0002** |
| Hyperlipidemia | 0.99(0.73-1.35) | .986 |
| Diabetes mellitus | 0.97(0.71-1.33) | .890 |
| AF at echo | 1.15(0.85-1.55) | .356 |
| Antiplatelet | 1.47(1.11-1.96) | **.006** |
| Anticoagulant | 1.17(0.84-1.62) | .351 |
| RASi or ARNi | 0.98(0.74-1.30) | .908 |
| Beta-blocker | 0.72(0.54-0.96) | **.026** |
| Calcium channel blocker | 0.61(0.42-0.88) | **.006** |
| MRA | 1.43(1.07-1.90) | **.014** |
| Statin | 1.36(1.01-1.83) | **.046** |
| Diuretics | 1.41(1.02-1.94) | **.029** |
| Digoxin | 1.08(0.78-1.50) | .607 |
| MR phenotype (AFMR as reference) |  |  |
| VFMRst | 1.53(1.04-2.26) | **.030** |
| VFMRat | 1.62(1.04-2.52) | **.029** |
| LVEF, per 10% | 0.72(0.63-0.82) | **<.001** |
| A4C-LVLS | 0.91(0.87-0.94) | **<.0001** |
| LVEDD, mm | 1.02(1.00-1.04) | **.002** |
| LVESD, mm | 1.02(1.01-1.04) | **<.001** |
| LVESDi, mm/m^2^ | 1.04(1.02-1.06) | **<.001** |
| LVESVi, ml/m^2^ | 1.01(1.01-1.02) | **<.001** |
| MR-VC, mm | 1.15(1.03-1.28) | **.013** |
| Mitral annulus index, mm/m^2^ | 0.99(0.95-1.04) | .940 |
| TRPG | 1.01(0.99-1.01) | .195 |
| TR ≥ moderate-severe | 0.96(0.68-1.36) | .850 |
| LAVi per 5 ml/m^2^ | 1.01(0.99-1.03) | .190 |
| LASr | 0.95(0.92-0.97) | **<.001** |
| RV-FAC | 0.96(0.95-0.98) | **<.001** |
| RV Function | 1.49(1.30-1.70) | **<.001** |
| Time-dependent procedures | 1.46(1.01-2.11) | **.043** |

See Table 1 and 2 for abbreviations and definitions

**Supplemental Figure 2. Incremental analysis for the endpoint of CVD/HTx/LVAD at 5-year follow-up**

**
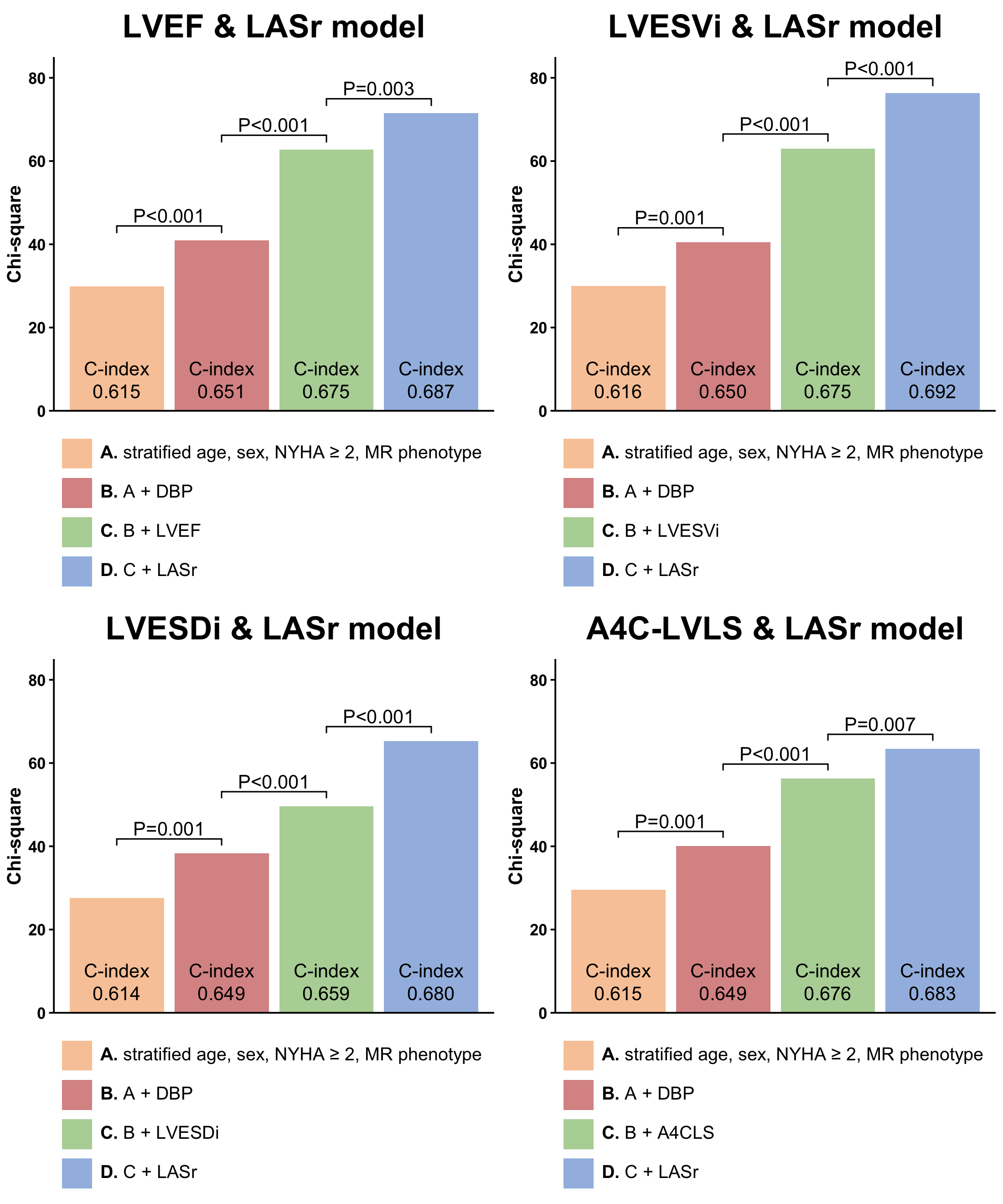
**

**Supplemental Table 3. Univariable determinants of ACD/HTx/LVAD (n=356) during 5-year follow-up**

|  | **HR (95% CI)** | P |
| --- | --- | --- |
| Age per year | 1.02(1.01-1.03) | **<.001** |
| CCI | 1.15(1.10-1.20) | **<.001** |
| Female | 0.74(0.60-0.91) | **.005** |
| SBP | 0.99(0.99-1.00) | .069 |
| DBP | 0.98(0.98-0.99) | **<.001** |
| NYHA ≥ II | 1.83(1.24-2.69) | **.001** |
| Hyperlipidemia | 0.91(0.72-1.14) | .402 |
| Diabetes mellitus | 1.13(0.91-1.42) | .270 |
| AF at echo | 0.99(0.79-1.24) | .923 |
| Antiplatelet | 1.48(1.20-1.82) | **<.001** |
| Anticoagulant | 0.95(0.73-1.22) | .671 |
| RASi or ARNi | 0.89(0.72-1.10) | .294 |
| Beta-blocker | 0.72(0.58-0.89) | **.001** |
| Calcium channel blocker | 0.75(0.58-0.97) | **.026** |
| MRA | 1.28(1.04-1.58) | **.023** |
| Statin | 1.13(0.90-1.42) | .284 |
| Diuretics | 1.22(0.97-1.52) | .088 |
| Digoxin | 0.95(0.74-1.21) | .668 |
| MR phenotype (AFMR as reference) |  |  |
| VFMRst | 1.52(1.15-2.02) | **.003** |
| VFMRat | 1.38(0.99-1.91) | .055 |
| LVEF, per 10% | 0.82(0.75-0.90) | **<.001** |
| A4C-LVLS | 0.93(0.91-0.96) | **<.001** |
| LVEDD, mm | 1.00(0.99-1.01) | .648 |
| LVESD, mm | 1.01(1.00-1.02) | **.029** |
| LVESDi, mm/m^2^ | 1.02(1.01-1.04) | **.007** |
| LVESVi, ml/m^2^ | 1.01(1.00-1.01) | **<.001** |
| MR-VC, mm | 1.05(0.96-1.14) | .261 |
| Mitral annulus index, mm/m^2^ | 0.99(0.96-1.02) | .506 |
| TRPG | 1.00(0.99-1.01) | .214 |
| LAVi per 5 ml/m^2^ | 1.00(0.99-1.01) | .639 |
| LASr, % | 0.97(0.95-0.99) | **<.001** |
| RV-FAC | 0.98(0.97-0.99) | **.002** |
| RV function | 1.21(1.09-1.34) | **<.001** |
| Time-dependent procedures | 1.18(0.89-1.58) | .255 |

See Table 1 for abbreviations and definitions

ACD, All-cause death.

**Supplemental Table 4. Multivariable determinants of ACD/HTx/LVAD (n=356) at 5-year follow-up***

|  | **LVEF model** | | **LVESDi model** | | **LVESVi model** | | **A4C-LVLS model** | |
| --- | --- | --- | --- | --- | --- | --- | --- | --- |
|  | HR (95% CI) | P | HR (95% CI) | P | HR (95% CI) | P | HR (95% CI) | P |
| MR phenotype (Ref: AFMR) |  |  |  |  |  |  |  |  |
| VFMRst | 1.02(0.67-1.56) | .911 | 1.15(0.79-1.67) | .466 | 1.07(0.75-1.54) | .692 | 1.19(0.85-1.66) | .313 |
| VFMRat | 0.98(0.64-1.51) | .943 | 1.10(0.74-1.63) | .630 | 1.02(0.69-1.51) | .907 | 1.14(0.79-1.65) | .484 |
| Female | 0.87(0.68-1.10) | .241 | 0.83(0.65-1.05) | .121 | 0.87(0.68-1.10) | .236 | 0.84(0.67-1.06) | .148 |
| CCI | 1.12(1.07-1.17) | **<.001** | 1.12(1.07-1.17) | **<.001** | 1.12(1.07-1.18) | **<.001** | 1.12(1.07-1.17) | **<.001** |
| NYHA ≥ II | 1.38(0.88-2.16) | .164 | 1.33(0.85-2.06) | .208 | 1.39(0.89-2.15) | .146 | 0.94(0.91-0.98) | .118 |
| DBP, per 5 mmHg | 0.94(0.91-0.98) | **.005** | 0.94(0.91-0.98) | **.004** | 0.95(0.91-0.99) | **.008** | 0.92(0.88-0.97) | **.002** |
| RV-FAC | 0.99(0.98-1.00) | .186 | 0.99(0.97-1.00) | **.049** | 0.99(0.97-1.00) | .073 | 0.99(0.97-1.00) | **.040** |
| LASr | 0.98(0.96-1.00) | **.048** | 0.98(0.96-1.00) | **.020** | 0.98(0.96-1.00) | **.019** | 0.98(0.96-1.00) | **.038** |
| LVEF, per 10% | 0.87(0.75-1.01) | .071 | — | — | — | — | — | — |
| LVESDi, per mm/m^2^ | — | — | 1.01(0.99-1.03) | .406 | — | — | — | — |
| LVESVi, per 10ml/m^2^ | — | — | — | — | 1.04(1.00-1.09) | .055 | — | — |
| A4C-LVLS >9.3% | — | — | — | — | — | — | 0.87(0.68-1.12) | .295 |
| Time-dependent procedures# | 1.22(0.89-1.68) | .220 | 1.12(0.82-1.54) | .464 | 1.17(0.85-1.60) | .327 | 1.12(0.82-1.52) | .493 |

See Table 1 and 2 for abbreviations and definitions

* Models also adjusted for stratified age (cutoff at 70 years old), beta blocker, antiplatelet, CCB, and MRA.

**Supplemental Table 5. Head-to-head comparison of A4C-LVLS and LVEF for the endpoint of CVD/HTx/LVAD at 5-year follow-up***

|  | LVEF + A4C-LVLS | | LVEF + A4C-LVLS >9.3% | |
| --- | --- | --- | --- | --- |
|  | HR (95% CI) | P | HR (95% CI) | P |
| MR phenotype (Ref: AFMR) |  |  |  |  |
| VFMRst | 0.66(0.38-1.14) | .138 | 0.66(0.37-1.15) | .140 |
| VFMRat | 0.84(0.48-1.48) | .554 | 0.86(0.49-1.51) | .595 |
| Female | 0.75(0.54-1.04) | .084 | 0.74(0.53-1.03) | .075 |
| NYHA ≥ II | 2.04(0.94-4.45) | .073 | 2.00(0.92-4.36) | .080 |
| DBP, per 5 mmHg | 0.93(0.88-0.98) | **.006** | 0.92(0.88-0.98) | **.005** |
| RV-FAC | 0.99(0.96-1.01) | .148 | 0.99(0.97-1.01) | .171 |
| LVEF, per 10% | 0.77(0.61-0.96) | **.022** | 0.76(0.62-0.94) | **.012** |
| A4C-LVLS, % | 0.96(0.91-1.01) | .130 | — | — |
| A4C-LVLS >9.3% | — | — | 0.68(0.47-0.99) | **.042** |
| Time-dependent procedures | 1.61(1.06-2.43) | **.024** | 1.56(1.03-2.35) | **.037** |

See Table 1 and 2 for abbreviations and definitions

*Both models also adjusted for stratified age (cutoff at 70 years old), MR-VC, statin, beta blocker, antiplatelet, CCB, MRA, and diuretics.

**Supplemental Table 6. Univariable determinants of CVD/HTx/LVAD in 3 subgroups in 5-year follow-up**

|  | **AFMR N=151, 33 endpoints** | | **VFMRat N=172, 51 endpoints** | | **VFMRst N=376, 109 endpoints** | |
| --- | --- | --- | --- | --- | --- | --- |
|  | HR (95% CI) | P | HR (95% CI) | P | HR (95% CI) | P |
| Age per year | 1.04(1.01-1.08) | **.007** | 1.01(0.99-1.03) | .405 | 1.01(0.99-1.02) | .236 |
| CCI | 0.95(0.77-1.14) | .587 | 1.03(0.91-1.15) | .640 | 1.08(0.99-1.17) | .095 |
| Female | 0.65(0.32-1.30) | .231 | 0.56(0.31-1.02) | **.049** | 0.65(0.43-0.97) | **.029** |
| SBP | 0.99(0.97-1.01) | .250 | 0.98(0.97-0.99) | **.016** | 0.99(0.99-1.00) | .233 |
| DBP | 0.99(0.96-1.01) | .231 | 0.99(0.97-1.01) | .185 | 0.98(0.96-0.99) | **.001** |
| NYHA ≥ II | 1.84(0.71-4.76) | .181 | 2.70(0.66-11.09) | .107 | 3.88(1.23-12.24) | **.004** |
| Hyperlipidemia | 0.81(0.33-1.97) | .630 | 0.44(0.23-0.86) | **.010** | 1.40(0.95-2.07) | **.093** |
| Diabetes mellitus | 0.17(0.02-1.25) | **.019** | 0.72(0.39-1.33) | .278 | 1.18(0.80-1.74) | .401 |
| AF at echo | 2.19(0.85-5.68) | .078 | 2.12(1.20-3.75) | **.013** | 1.09(0.66-1.80) | .747 |
| Antiplatelet | 1.96(0.96-3.99) | .075 | 0.87(0.50-1.51) | .625 | 1.59(1.07-2.35) | **.019** |
| Anticoagulant | 1.13(0.56-2.27) | .739 | 1.80(0.99-3.29) | .067 | 1.16(0.71-1.88) | .558 |
| RASi or ARNi | 0.94(0.47-1.89) | .862 | 0.98(0.57-1.70) | .950 | 0.96(0.66-1.40) | .849 |
| Beta-blocker | 0.77(0.39-1.53) | .466 | 0.75(0.43-1.29) | .298 | 0.71(0.49-1.03) | .071 |
| Calcium channel blocker | 0.57(0.23-1.38) | .186 | 0.46(0.20-1.08) | **.049** | 0.71(0.44-1.13) | .135 |
| MRA | 1.25(0.61-2.54) | .547 | 2.18(1.26-3.79) | **.006** | 1.22(0.83-1.78) | .316 |
| Statin | 1.79(0.81-4.00) | .175 | 0.81(0.44-1.50) | .500 | 1.49(1.01-2.18) | **.045** |
| Diuretics | 0.73(0.37-1.45) | .368 | 1.99(0.97-4.09) | **.044** | 1.60(0.97-2.30) | .058 |
| Digoxin | 0.85(0.39-1.82) | .665 | 1.80(0.98-3.29) | .068 | 0.99(0.64-1.54) | .967 |
| LVEF, per 10% | 1.07(0.73-1.61) | .744 | 0.56(0.43-0.74) | **<.001** | 0.70(0.55-0.89) | **.003** |
| A4C-LVLS | 1.02(0.94-1.11) | .626 | 0.84(0.77-0.91) | **<.001** | 0.89(0.84-0.94) | **<.001** |
| LVEDD, mm | 0.99(0.96-1.04) | .982 | 1.04(1.01-1.08) | **.016** | 1.02(1.00-1.04) | .093 |
| LVESD, mm | 1.01(0.96-1.04) | .804 | 1.05(1.02-1.08) | **.001** | 1.02(1.00-1.04) | **.022** |
| LVESDi, mm/m^2^ | 1.03(0.96-1.09) | .388 | 1.08(1.04-1.13) | **<.001** | 1.02(0.99-1.05) | .239 |
| LVESVi, ml/m^2^ | 1.00(0.98-1.02) | .897 | 1.01(1.01-1.02) | **<.001** | 1.01(1.00-1.02) | **.001** |
| MR-VC, mm | 1.39(1.08-1.78) | **.012** | 1.17(0.93-1.45) | .185 | 1.07(0.91-1.25) | .405 |
| Mitral annulus index, mm/m^2^ | 1.09(0.99-1.20) | .065 | 0.99(0.90-1.08) | .865 | 1.00(0.93-1.07) | .956 |
| TRPG | 1.00(0.98-1.03) | .791 | 1.00(0.99-1.02) | .647 | 1.01(1.00-1.02) | .161 |
| TR ≥ moderate-severe | 1.73(0.87-3.42) | .119 | 1.03(0.51-2.04) | .935 | 0.78(0.46-1.36) | .380 |
| LAVi per 5 ml/m^2^ | 1.02(1.00-1.05) | .082 | 1.05(0.99-1.11) | .101 | 1.04(0.99-1.09) | .115 |
| LASr | 0.97(0.91-1.02) | .237 | 0.90(0.84-0.95) | **<.001** | 0.96(0.93-0.99) | **.004** |
| RV-FAC | 0.98(0.94-1.02) | .293 | 0.96(0.93-0.99) | **.012** | 0.97(0.95-0.99) | **.016** |
| RV function | 1.54(1.05-2.18) | **.028** | 1.54(1.20-1.98) | **.001** | 1.41(1.17-1.70) | **<.001** |
| Time-dependent procedures | 0.56(0.20-1.61) | .285 | 0.83(0.37-1.85) | .648 | 2.65(1.68-4.17) | **<.001** |

See Table 1 and 2 for abbreviations and definitions

**Supplemental Table 7. Multivariable determinants of CVD/HTx/LVAD in 3 subgroups at 5-year follow-up**

|  | **1^st^ model** | | **2^nd^ model** | | **3^rd^ model** | |
| --- | --- | --- | --- | --- | --- | --- |
|  | HR (95% CI) | P | HR (95% CI) | P | HR (95% CI) | P |
|  | **AFMR* (N=151, 33 endpoints)** | | | | | |
| LAVi per 5 ml/m^2^ | 1.01(1.00-1.01) | **.041** | 1.01(1.00-1.01) | **.008** | 1.01(1.00-1.01) | **.009** |
| MR-VC, mm | 1.43(1.09-1.87) | **.009** | — | — | — | — |
| RV function | — | — | 1.68(1.15-2.46) | **.008** | — | — |
| RV-FAC | — | — | — | **—** | 0.97(0.93-1.01) | .157 |
|  | **VFMRat (N=172, 51 endpoints)** | | | | | |
| Female | 0.72(0.39-1.34) | .302 | 0.72(0.39-1.33) | .292 | 0.69(0.37-1.27) | .233 |
| AF at echo | 1.63(0.89-2.97) | .114 | 1.96(1.08-3.57) | **.027** | 1.92(1.07-3.46) | **.029** |
| LASr, % | 0.93(0.87-0.99) | **.045** | 0.94(0.88-1.00) | .062 | 0.95(0.89-1.02) | .148 |
| RV-FAC | 0.99(0.96-1.04) | .876 | 0.99(0.95-1.03) | .575 | 0.99(0.95-1.03) | .569 |
| LVEF, per 10% | 0.68(0.49-0.93) | **.016** | — | — | — | — |
| LVESVi, per 10 ml/m^2^ | — | — | 1.12(1.04-1.21) | **.005** | — | — |
| A4C-LVLS, % | — | — | — | — | 0.89(0.79-0.99) | **.030** |
|  | **VFMRst (N=376, 109 endpoints)** | | | | | |
| Female | 0.74(0.49-1.13) | .159 | 0.70(0.46-1.07) | .095 | 0.68(0.45-1.04) | .077 |
| DBP, mmHg | 0.98(0.97-0.99) | **.004** | 0.98(0.97-0.99) | **.010** | 0.98(0.97-0.99) | **.004** |
| NYHA ≥ II | 3.15(0.77-12.96) | .112 | 3.00(0.73-12.31) | .128 | 3.24(0.79-13.29) | .102 |
| RV-FAC | 0.99(0.97-1.03) | .982 | 0.99(0.97-1.02) | .589 | 0.99(0.96-1.01) | .366 |
| LASr, % | 0.97(0.94-1.01) | .121 | 0.96(0.93-0.99) | **.038** | 0.99(0.94-1.03) | .491 |
| LVEF, per 10% | 0.74(0.56-0.98) | **.034** | — | — | — | — |
| LVESVi, per 10 ml/m^2^ | — | — | 1.09(1.03-1.16) | **.006** | — | — |
| A4C-LVLS, % | — | — | — | — | 0.93(0.86-0.99) | **.041** |
| Time-dependent procedures | 2.51(1.55-4.07) | **<.001** | 2.35(1.46-3.77) | **<.001** | 2.30(1.43-3.69) | **.001** |

See Table 1 and 2 for abbreviations and definitions

*Models were also adjusted for stratified age (cutoff at 70 years old)
